# Management of Discoloured Anterior Teeth With Radicular Cyst: A Case Report

**DOI:** 10.7759/cureus.45536

**Published:** 2023-09-19

**Authors:** Paridhi Agrawal, Pradnya Nikhade, Aditya Patel, Shweta Sedani, Jay Bhopatkar

**Affiliations:** 1 Department of Conservative Dentistry and Endodontics, Sharad Pawar Dental College and Hospital, Datta Meghe Institute of Higher Education and Research (DMIHER), Wardha, IND

**Keywords:** apicoectomy, walking bleach, radicular cyst, platelet-rich-fibrin, nanocrystalline hydroxyapatite, intracoronal bleaching, discolored tooth

## Abstract

Dental trauma often has tooth discoloration and periapical lesion as its sequelae. Intracoronal bleaching restores the aesthetics, while a retrograde approach is required for non-healing lesions. A patient with discolored teeth, draining sinus, tenderness and a periapical lesion on the radiograph was treated initially with conventional root canal therapy and walking bleach technique. After four months, the sinus tract reappeared and on cone-beam computed tomography (CBCT) examination, a large periapical lesion with loss of buccal cortical plate was seen. A minimally invasive retrograde cystic enucleation, apicectomy, and filling with biodentine were then performed. The defect area was filled with synthetic nanocrystalline hydroxyapatite granules combined with platelet-rich-fibrin. Follow-ups after one, three, and six months were taken. The six-month CBCT revealed complete bone healing. Non-vital bleaching yields acceptable cosmetic results. Additionally, if the conventional procedures are not successful in treating radicular cyst, apical surgery must be the ultimate choice.

## Introduction

The field of aesthetics is becoming steadily important, especially in relation to having an attractive smile, as it can substantially affect an individual's psychological and social well-being by influencing their perception of their own appearance. The effect of discolored teeth is even more apparent when there is a single tooth that appears different in color, as it stands out more in comparison to the other teeth [1]. Discoloration of a non-vital tooth can be an outcome of a variety of factors, including dental trauma, the presence of dead tissue or debris in the dentinal tubules and pulp horns, inadequate irrigation during treatment, or filling materials present in the pulp chamber or on the chamber walls. Dental trauma typically affects the dentoalveolar region and can result in damage to the tooth and the hard and soft tissues encompassing it. Common consequences of injuries from dental trauma include necrosis of the pulp leading to tooth discoloration, apical periodontitis, and cystic changes [2].

Internal tooth bleaching is a cost-effective, minimally invasive, and proficient method for treating discolored teeth that have undergone endodontic treatment and have an intact clinical crown. This procedure is fairly simple and conservative. There are diverse techniques used for internal tooth bleaching, whilst the walking-bleach technique is the most common. This technique was first introduced by Spasser in 1961 [3]. It often involves using a combination of sodium perborate and hydrogen peroxide to bleach the discolored tooth. This mixture is inserted into the pulp chamber and left for a specific period of time before being refreshed by the dentist periodically till the desired shade is obtained. The effectiveness of the bleaching process depends on several factors, including the concentration of the bleaching agent used, the ability of the agent to penetrate the chromophore molecules, and the duration and frequency of the contact between the agent and the chromophore molecules [4].

A radicular cyst is an inflammatory odontogenic cyst. It is typically caused by the activation and growth of epithelial remnants (known as cell rests of Malassez) following dental caries or trauma. This type of cyst is often found at the root tips of the affected teeth or along the lateral side when linked with an accessory or lateral canal [5]. Conventional root canal therapy is typically the primary treatment for managing periapical lesions, including cysts. Studies have proven that cysts and other large periapical lesions can reciprocate well to non-surgical treatment with calcium hydroxide intracanal medicament [6]. However, when a conventional root canal procedure is unachievable or has failed, periapical surgery may be contemplated as a viable alternative. In a retrospective observational study, it was identified that the predominant treatment for radicular cysts was enucleation followed by apicoectomy [7].

This article describes the successful management of a discolored non-vital tooth associated with a radicular cyst using conventional root canal treatment, walking bleach technique, cystic enucleation, and nanocrystalline hydroxyapatite bone graft and platelet-rich fibrin (PRF) membrane.

## Case presentation

**Figure 1:**
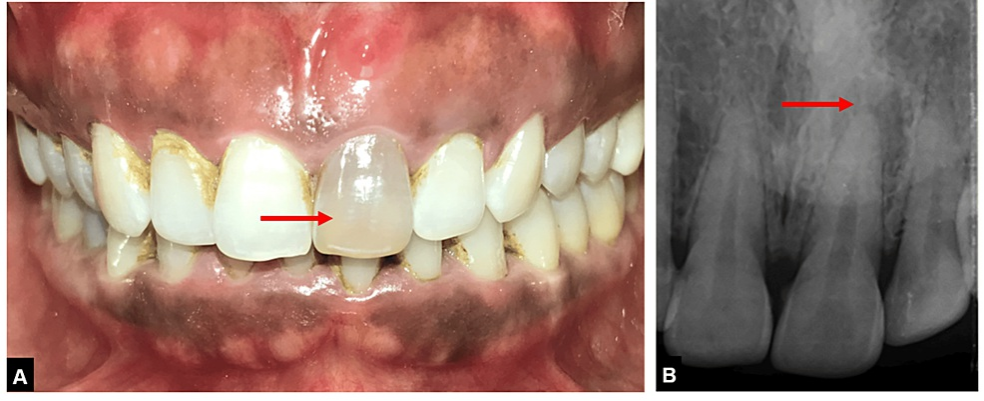
Pre-operative images (A) Clinical image showing draining sinus tract and discolored tooth with 21, (B) Radiograph showing periapical lesion with 21.

A 32-year-old patient presented to the Department of Conservative Dentistry and Endodontics with a chief complaint of a discolored tooth and pus discharge in the upper front region of her jaw. The patient reported a history of trauma (self-fall) in the same region eight years prior, after which the patient experienced mild pain that was relieved by taking analgesics. One year after the trauma, the patient noticed a small pink spot on the affected tooth that gradually grew larger and darker. This was accompanied by intermittent pus discharge, which occasionally caused mild pain. The patient did not report any associated swelling. There was no contributing medical history present. On intraoral examination, discolored tooth and associated draining sinus tract were seen with 21 (Figure 1A). Vertical tenderness on percussion was negative. Furthermore, the tooth showed no response to thermal and electric pulp tests decisive of being nonvital. On radiographic investigation, a periapical radiolucent shadow with a diffuse border was seen associated with 21 suggestive of periapical abscess with 21 (Figure 1B). It was chosen to perform a conventional root canal procedure with 21 subsequent to intracoronal bleaching with 21. The patient gave consent with knowledge of the treatment, and the procedure commenced.

Protocol for endodontic treatment

**Figure 2:**
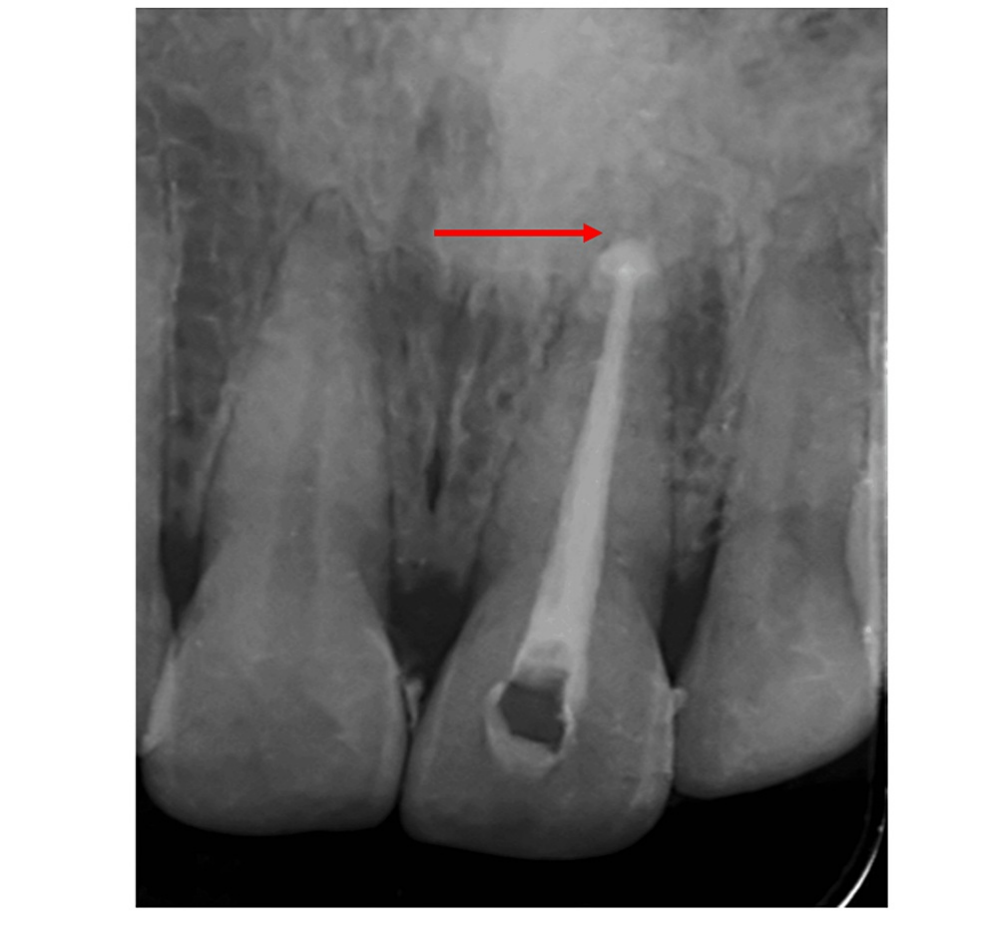
Post-operative image after obturation with 21.

Access opening was performed with 21 and working length was calculated with the aid of an apex locator and then verified radiographically. Using a K-file the tooth was shaped in a step-back manner to an apical file size of #60. The canal was irrigated copiously and two sittings of calcium hydroxide intracanal medicament dressing with a week interval was given with 21. Once the canal was completely dry and the patient was asymptomatic, mastercone fit was evaluated and obturation was performed with 21 using the cold lateral compaction technique (Figure 2).

Protocol for walking bleach technique

**Figure 3:**
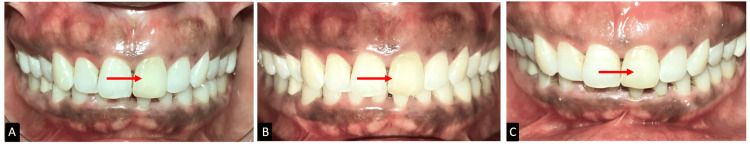
Post-bleaching images (A) Immediate post-operative bleaching results after three applications of bleaching agent, (B) one-month follow-up image, (C) four-month follow-up image.

In the successive visit, the tooth was prepared for intracoronal bleaching. A portion of the gutta-percha that was 2mm below the cementoenamel junction level was removed, and a mechanical barrier made of glass ionomer cement was placed. A creamy paste was created by mixing sodium tetrahydrate perborate and 10% carbamide peroxide, which was then placed in the pulp chamber. The cavity was sealed using temporary cement. This procedure was repeated three times at a one-week interval until the desired tooth whitening results were achieved (Figure 3A). Afterward, the patient was scheduled for follow-up appointments at one, three, six, and nine months during which it was noted that the achieved tooth shade remained constant with 21 (Figure 3B, 3C).

Recurrence of symptoms with 21: a CBCT investigation

**Figure 4:**
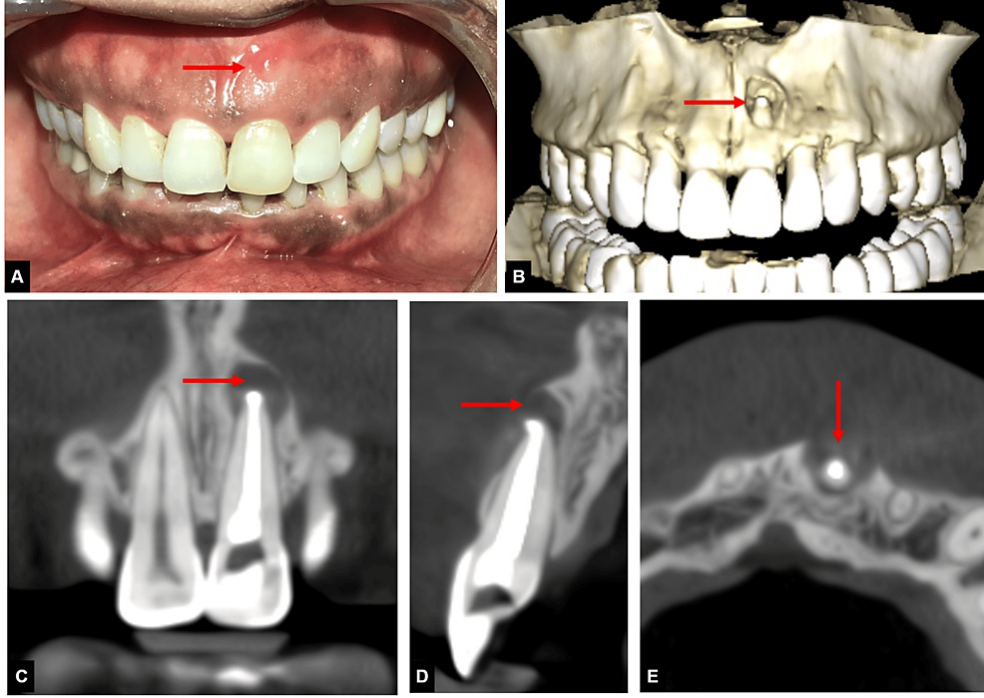
Images showing recurrence of symptoms with 21 (A) Image showing recurrence of sinus tract four months following obturation with 21, (B) CBCT image showing large periapical lesion with perforation of labial cortical plate with 21, (C) CBCT coronal view showing large periapical lesion with 21, (D) Axial CBCT view showing labial cortical plate perforation with 21, (E) sagittal CBCT view showing perforation of labial cortical plate. CBCT: Cone-beam computed tomography

After four months following the treatment, the patient returned with recurring symptoms, including a draining sinus tract associated with tooth 21 (Figure 4A). A CBCT assessment was performed and revealed a well-defined radiolucent shadow measuring 6.09 mm mediolaterally x 3.72 mm anteroposteriorly x 7.34 mm craniocaudally, which was associated with the periapical region of tooth 21. Additionally, there was a loss of the buccal cortical plate (Figure 4B-4E). The decision was made to perform a retrograde curettage of the periapical area, followed by apicoectomy and retrograde filling with tooth 21. The surgical procedure was explained to the patient and informed consent was obtained. All the necessary hematological examinations were done, and the patient was considered fit for the surgery and was recalled the next day.

Surgical treatment protocol

**Figure 5:**
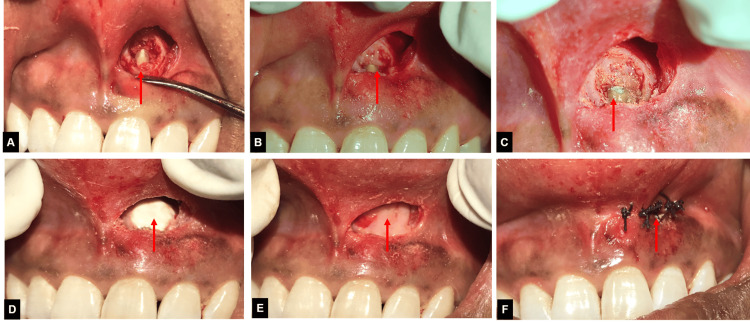
Images showing apicoectomy procedure (A) Image showing granulation tissue in periapical area and exposed root surface with 21, (B) Image showing granulation tissue debridement and apicoectomy with 21, (C) Image showing root-end filling using biodentine with 21, (D) Image showing placement of nanocrystalline hydroxyapatite granules in the defect site, (E) Image showing placement of platelet-rich-fibrin membrane in the defect site, (F) Image showing repositioned and sutured flap.

Lignocaine 2% with adrenaline 1:80,000 was administered at the surgical site, and a horizontal releasing incision was made at the mucogingival junction with respect to 21 and 22 region. Access to the defect was gained after elevating a full-thickness mucoperiosteal flap, the periapical area was completely curetted, the granulation tissue was removed, and the enucleation of the cystic lesion was done, and a specimen was taken for biopsy and sent for histopathological analysis (Figure 5A). Following that apicoectomy, root-end preparation, and retrograde filling with Biodentine were done with 21 (Figure 5B, 5C). Thereafter, 10 ml of the patient’s blood was withdrawn, transferred to a test tube, and centrifuged for 10 minutes at 3000 rpm to obtain a clear PRF. This PRF clot was taken out and compressed between two sterile gauzes for the preparation of the PRF membrane. The debrided cystic cavity was filled with a nanocrystalline hydroxyapatite bone graft (particle size 40-50 nanometers, Sybograf, Eucare Pharmaceuticals Private Limited, Chennai, India) and covered with a PRF membrane (Figure 5D, 5E). The flap was repositioned, and suturing was performed (Figure 5F).

**Figure 6:**
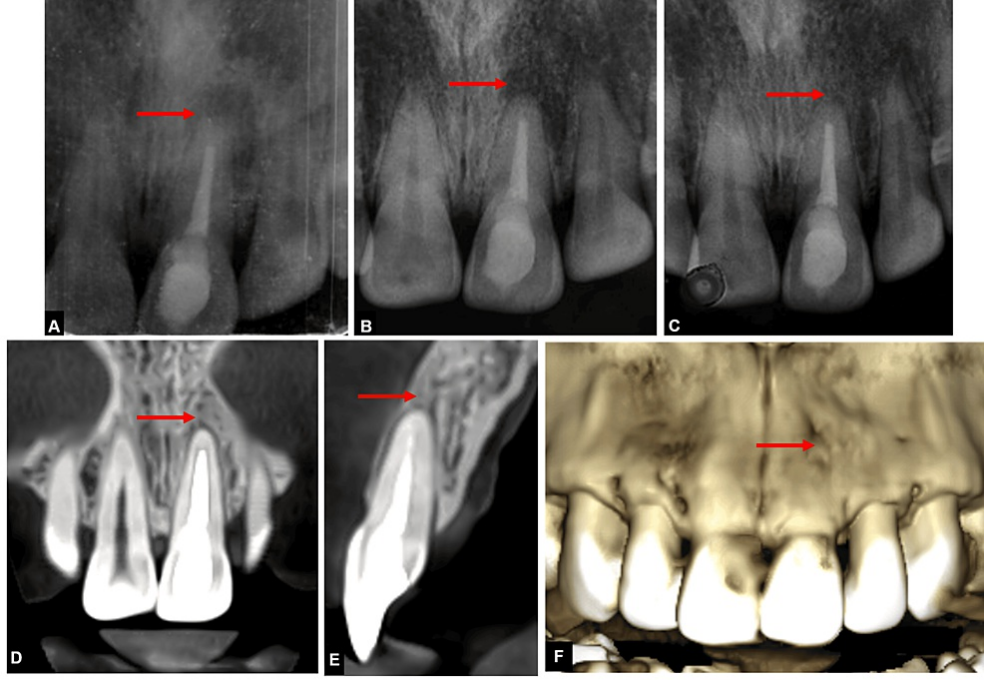
Post-operative and follow-up radiographs (A) Immediate post-operative radiograph after apicoectomy with 21, (B) one-month follow-up radiograph, (C) three-month follow-up radiograph, (D) coronal CBCT view showing healed periapical lesion with 21, (E) axial CBCT view showing healed periapical lesion with 21, (F) six-month follow-up CBCT image showing healed periapical lesion. CBCT: Cone-bone computed tomography

An immediate postoperative periapical radiograph was taken (Figure 6A). The biopsy taken during the surgery confirmed the presence of a radicular cyst. The patient was then evaluated at three, and six-month follow-ups during which complete bone healing was seen, and no symptoms such as discomfort, pain, or inflammation were noted (Figure 6B-6F).

## Discussion

Trauma-induced internal pulpal bleeding may spread blood constituents inside the dentinal tubules, resulting in discoloration of the tooth. Then, during hemolysis, iron is released through the blood breakdown products like haemosiderin, haemin, haematin, and haematoid. With the help of hydrogen sulfide created by bacteria, iron can be changed into black ferric sulfide, which stains the teeth [8]. The minimally invasive process of non-vital bleaching used to restore the aesthetics of the discolored non-vital teeth traditionally utilizes 30% hydrogen peroxide along with sodium perborate, but there is an increased risk of external cervical root resorption. The precise mechanism of resorption in bleached teeth is still not understood well. It has been hypothesized that hydrogen peroxide directly triggers an inflammatory resorption process by diffusing through the periodontal ligament, cementum, and dentinal tubules before reaching the periradicular bone [9]. Due to its efficiency and low levels of extraradicular diffusion, carbamide peroxide is the preferred substance for intracoronal bleaching. In an in vitro study, it was concluded that sodium perborate mixed with 10-35% carbamide peroxide produced better results [10]. Hence, a combination of carbamide peroxide and sodium perborate was used and the desired results after bleaching were obtained.

A radicular cyst is an inflammatory odontogenic cyst that originates after a persistent periapical granuloma and proliferation of cell remnants of Malassez. These inflammatory lesions cause resorption of the bone. They can grow significantly and exhibit symptoms when infected or because of neural compression. They typically range in size from 0.5 to 1.5 cm [11]. The size of the cyst in our case was approximately 0.7 cm. The preferred method for treating radicular cyst may depend on a number of variables, including the extent of the lesion and its clinical characteristics, the involvement of significant anatomical structures, patient preferences, and systemic health. The majority of radicular cysts can be treated using standard root canal therapy which was our primary plan. According to research, calcium hydroxide must be used for at least two weeks in order to have an antimicrobial impact and to reduce exudate [12]. Hence we treated the root canal across a number of sessions using a provisional calcium hydroxide intracanal medicament. In our instance, unanticipatedly the conventional root canal procedure was unsuccessful. So, we decided to perform apical surgery as this is the definitive procedure for the treatment of radicular cyst. This also has the added benefit of enabling excisional biopsy for histological evaluation to confirm the diagnosis. A histopathological diagnosis of a radicular cyst was concluded in our case.

After periapical surgery and cyst enucleation, using a bone graft helps hasten the process of bone growth in the affected area. In order to make way for the new bone, the bone graft eventually dissolves and serves as a foundation for new bone formation. Additionally, bone grafts can function as an osteoconductive material that encourages the migration of osteoprogenitor cells stabilizes blood clotting, and speeds up bone healing [13]. Nanocrystalline hydroxyapatite (NCHA) bone graft was placed in this case. It was concluded in a study that the properties of high cell adhesion, the viability of osteoblast, and osteoconductivity innate with NCHA make it an excellent bone defect regeneration scaffold [14]. Because of its beneficial sealing ability with the fibrin adhesive, PRF has demonstrated effectiveness in minimizing postoperative hematoma along with its potential in promoting healing. In a study, it was found that a substantially high healing rate (around 92%) was seen with PRF after six months [15]. Hence, a combination of NHCA and PRF was used in our case which has shown evident results.

## Conclusions

Non-vital bleaching is a quick, reliable, and economical procedure that yields acceptable cosmetic results. The brightening discolored teeth using the walking bleach technique can be seen as a fairly simple, efficient, and quick technique employed in clinical preference for both the patient and the clinician in today's world of cosmetic dentistry which seeks aesthetics and concurrently a minimally invasive approach for the fruitful restorative outcome. The endodontist must, however, carefully choose appropriate patients and steer clear of any potential problems both during and after the treatment. Moreover, conventional endodontic therapy ought to be the initial choice for treating a radicular cyst. If the conventional procedures are not successful in treating a radicular cyst, apical surgery must be the ultimate choice. Scaffolds can be utilized to achieve prompt results as well.
